# Experimental Models of Cognitive Impairment for Use in Parkinson’s Disease Research: The Distance Between Reality and Ideal

**DOI:** 10.3389/fnagi.2021.745438

**Published:** 2021-11-29

**Authors:** Yaohua Fan, Jiajun Han, Lijun Zhao, Chunxiao Wu, Peipei Wu, Zifeng Huang, Xiaoqian Hao, YiChun Ji, Dongfeng Chen, Meiling Zhu

**Affiliations:** ^1^Traditional Chinese Medicine Innovation Research Center, Shenzhen Hospital of Integrated Traditional Chinese and Western Medicine, Guangzhou University of Chinese Medicine, Shenzhen, China; ^2^Guangzhou University of Chinese Medicine, Guangzhou, China; ^3^Shenzhen Bao’an Traditional Chinese Medicine Hospital, Guangzhou University of Chinese Medicine, Shenzhen, China

**Keywords:** Parkinson’s disease, cognitive impairment, experimental model, toxins, transgenic animal, limitations

## Abstract

Parkinson’s disease (PD) is the second most common neurodegenerative disease. Cognitive impairment is one of the key non-motor symptoms of PD, affecting both mortality and quality of life. However, there are few experimental studies on the pathology and treatments of PD with mild cognitive impairment (PD-MCI) and PD dementia (PDD) due to the lack of representative models. To identify new strategies for developing representative models, we systematically summarized previous studies on PD-MCI and PDD and compared differences between existing models and diseases. Our initial search identified 5432 articles, of which 738 were duplicates. A total of 227 articles met our inclusion criteria and were included in the analysis. Models fell into three categories based on model design: neurotoxin-induced, transgenic, and combined. Although the neurotoxin-induced experimental model was the most common type that was used during every time period, transgenic and combined experimental models have gained significant recent attention. Unfortunately, there remains a big gap between ideal and actual experimental models. While each model has its own disadvantages, there have been tremendous advances in the development of PD models of cognitive impairment, and almost every model can verify a hypothesis about PD-MCI or PDD. Finally, our proposed strategies for developing novel models are as follows: a set of plans that integrate symptoms, biochemistry, neuroimaging, and other objective indicators to judge and identify that the novel model plays a key role in new strategies for developing representative models; novel models should simulate different clinical features of PD-MCI or PDD; inducible α-Syn overexpression and SH-SY5Y-A53T cellular models are good candidate models of PD-MCI or PDD.

## Introduction

Cognitive impairment, one of the key non-motor comorbid symptoms of PD, is not only a risk factor for early mortality but also has a greater impact on quality of life than motor symptoms (Oosterveld et al., 2015; Saredakis et al., 2019). Cognitive impairment in PD can be divided based on severity into mild cognitive impairment (PD-MCI) and dementia (PDD). PD-MCI, an intermediate cognitive stage on the continuum between normal cognitive function and dementia, can be identified in 15%–40% of PD patients at the time of diagnosis (Aarsland, 2016; Monastero et al., 2018). The prevalence of PD-MCI and PDD in the PD population is 40% and 30%, respectively (Aarsland and Kurz, 2010; Baiano et al., 2020). PDD occurs in 75%–90% of patients who have had PD for 10 years or more (Gratwicke et al., 2015). Understanding the pathophysiology of PD-MCI and PDD is critical to the development of disease-modifying treatments. Although only 5%–15% of PD cases are attributed to clear genetic factors, several studies have shown that genetic factors such as α-synuclein (α-Syn) variants play an important role in cognitive impairment (Ball et al., 2019; Chen et al., 2020). The 85%–95% remaining idiopathic cases of PD can be linked to environmental factors, such as heavy metals, pesticides, and illicit drugs, whose important role cannot be overlooked when studying cognitive impairment (Ball et al., 2019; Cariccio et al., 2019; Agnihotri and Aruoma, 2020). Systemic medical factors are also associated with PD cognitive impairment, including lower uric acid levels and the presence of an orthostatic heart rate or orthostatic hypotension (McDonald et al., 2016; Veselý et al., 2019; Tanaka et al., 2020). Gene–environment–medical history interactions may play a vital role in the pathogenesis of PD-MCI and PDD. Because the development of cognitive impairment in patients with PD is a multi-factor interaction, the heterogeneous pathophysiologic mechanisms underlying PD-MCI and PDD remain unclear. Recent studies have shown that inhibitors, amino acids, and actively targeted gold nanoparticle composites could improve the cognitive flexibility of experimental models. There are very few therapeutic options for PD patients with cognitive impairment due to the limited efficacy and significant side effects of therapeutic methods (Hsu et al., 2018; Byers et al., 2020; Liu L. et al., 2020; Wang et al., 2021). In order to identify risk factors, mechanisms, and treatment strategies for PD-MCI and PDD, research using appropriate experimental models is invaluable.

Experimental models, an important part of basic science research that emulates human physiology or pathology, are crucial to improving our understanding of PD-MCI and PDD pathophysiology and to the preclinical testing of novel therapeutics. Prior use of multitudinous PD experimental models has assessed the different symptoms and stages of PD and includes classical neurotoxin-induced, transgenic, and combined models (Kin et al., 2019). Some experimental PD models replicate PD cognitive impairment, which is very useful for studies on PD-MCI and PDD. These models include genetic and toxin-based exposure to a variety of animals (Prediger et al., 2010; Ba et al., 2019; Singh et al., 2019). However, each model exhibited varying degrees of motor and cognitive dysfunction symptoms or only produced mild symptoms of cognitive dysfunction alone because of the tangle modeling methods that were used. These experimental models, which were assessed with uneven behavioral tests such as the NOR, MWM, Y-maze test, T-maze test, and Barnes maze test demonstrated short-term or long-term spatial memory deficits, episodic-like memory deficits and recognition memory deficits (Huang et al., 2015; Singh et al., 2019; Ding et al., 2020). Regrettably, an ideal and representative experimental model that replicates all of the phenotypic and pathologic features of PD-MCI and PDD is still unavailable. However, each existing model has its own benefits and drawbacks that are important to consider when selecting an appropriate model based on the specific objectives of the study (Grandi et al., 2018). It is therefore vital to have a comprehensive understanding of the neuropathology of each model, permitting an accurate explanation of research results and a reasonable estimate of how well the model will correlate with human PD-MCI and PDD. In this review, we analyze trends in PD cognitive impairment modeling and discuss the features and limitations of current models based on the clinical features of PD-MCI and PDD.

## Clinical Features of Parkinson’s Disease Mild Cognitive Impairment and Parkinson’s Disease’s Disease Dementia

### Typical Symptoms

The two cardinal symptoms of PD-MCI and PDD are motor deficits (i.e., asymmetric resting tremor, bradykinesia, postural instability, and rigidity) and one or more cognitive deficits in attention, working memory, executive function, language, memory, visuo-constructive ability, and visuospatial function (Dubois et al., 2007; Litvan et al., 2012; Hayes, 2019). Unlike PD-MCI patients, whose symptoms are insufficient to interfere with functional independence, PDD is characterized by the impairment of two or more of the four cognitive domains and leads to more severe cognitive deficits that impair daily life based on the proposed criteria of the Movement Disorder Society Task Force (Table 1) (Dubois et al., 2007; Litvan et al., 2012). Representative experimental models of PD-MCI or PDD therefore, need to develop both motor and cognitive deficiency symptoms. Moreover, the cognitive dysfunction shown by the ideal model of PDD should be distinct from that of PD-MCI. We need to evaluate more than four cognitive domains through a variety of behavioral studies to clarify the degree of cognitive impairment of the ideal model and prove whether this ideal model represents PD-MCI or PDD.

**TABLE 1:** The clinical features of PD-MCI and PDD.

Clinical features		PD-MCI	PDD
Typical symptoms	Motor symptom	Any two of: 1. Asymmetric resting tremor 2. Asymmetric bradykinesia 3. Asymmetric postural instability 4. Asymmetric rigidity	
	Symptoms of cognitive impairment	Any one of: 1. Attention and working memory 2. Executive function 3. Language 4. Memory 5. Visuospatial function	Any two or more of: 1. Attention 2. Executive function 3. Visuo-constructive ability 4. Memory
Neuropathology		(Significant pathological heterogeneity) 1. α-Synucleinopathy in locus ceruleus, amygdala, cingulate gyrus and middle temporal gyrus, argyrophilic grains 2. A single neurofibrillary tangle in the hippocampal CA1 region 3. White matter rarefaction in the frontal lobe 4. Amyloid plaques and neurofibrillary tangles in the transentorhinal area 5. Thorn-shaped astrocytes characteristic of aging-related tau astrogliopathy in the subpial region of the amygdala	1. α-Synucleinopathy in the pigmented neuron of substantia nigra, amygdala, cortex and hippocampal CA2 region. 2. Tau pathology 3. Amyloid-β pathology
Biomarkers in biofluids	CSF	1. Low Aβ_42_ levels	1. Low Aβ_42_ levels 2. High levels of t-tau, p-tau and total α-Syn.
	Serum/plasma	1. GBA, LRRK2 and α-Syn mutations. 2. High α-synuclein level 3. Low level of EGF, GDNF, miR-29s and vitamin D	
Neuroimaging	MRI	1. A pattern of cortical volume loss in parietal, posterior and frontal cortices 2. Atrophy in the hippocampus	1. Extensive cortical atrophy in parietal, occipital, temporal and frontal cortices 2. Substantial atrophy in the hippocampus, including the parahippocampus, insula and cingulate gyrus
	PET	1. DAergic deficits in the striatum and the insula 2. Lower level of metabolic activity in the parietal, temporal, cingulate and frontal cortices	1. DAergic deficits in the striatum (caudate, putamen and pallidum), anterior cingulate and midbrain 2. Lower level of metabolic activity in the parietal, temporal, cingulate and frontal cortices 3. Loss of cholinergic function in the temporal, frontal and medial occipital cortices and thalamus

*PD-MCI, Parkinson’s disease mild cognitive impairment; Parkinson’s disease’s disease dementia; α-Syn, α-synuclein; Aβ_42_, amyloid-β 42; CSF, cerebrospinal fluid; GBA, glucosylceramidase; LRRK2, leucine-rich repeat serine/threonine-protein kinase 2; GDNF, glial cell line-derived neurotrophic factor; miR-29s, miRNA-29s; EGF, epidermal growth factor; MRI, magnetic resonance imaging; PET, positron emission tomography; DAergic, dopaminergic.*

### Neuropathology

The representative pathologic substrate of both PD-MCI and PDD mainly includes LBs and AD pathology. A longitudinal clinical study found that the representative types of pathologies that contribute to PD-MCI includes α-synucleinopathy in the locus coeruleus, amygdala, cingulate gyrus, and middle temporal gyrus, argyrophilic grains, a single neurofibrillary tangle in the hippocampal CA1 region, white matter rarefaction in the frontal lobe, amyloid plaques and neurofibrillary tangles in the transentorhinal area, and thorn-shaped astrocytes characteristic of aging-related tau astrogliopathy in the subpial region of the amygdala (Knox et al., 2020). However, there was significant heterogeneity in the clinical presentation of PD-MCI due to the differing impact of the individual disease pathology on each cognitive domain (Halliday et al., 2014; Knox et al., 2020).

The most persuasive evidence to date suggests that Lewy-type α-synucleinopathy plays a paramount role in PDD. α-synucleinopathy was found in the pigmented neurons of the SN, amygdala, cortex, and hippocampal CA2 region (Halliday et al., 2014; Liu et al., 2019). Recent studies found that a progressively decreased α-Syn pathologic load between the anterior periallocortical agranular toward the intermediate dysgranular and posterior isocortical granular insular subregions and an increase in activated microglia in the amygdala were significantly related to the extent of α-Syn pathology (Fathy et al., 2019; Kouli et al., 2020). A systematic review found that tau and amyloid-β pathologies independently contributed to the development of PDD (Smith et al., 2019). Moreover, the intricate causal relationship between PD and cerebral amyloid angiopathy, hippocampal sclerosis, small vessel disease, and other pathological factors remains unclear and therefore requires further research (Halliday et al., 2014; Hase et al., 2020).

### Biomarkers in Biofluids

The relationships between PD cognitive dysfunction and biomarkers in biofluids [i.e., cerebrospinal fluid (CSF), serum/plasma] have been studied in great detail (Johar et al., 2017). Biomarkers in the CSF could represent cognitive decline in PD due to their proximity to the central nervous system. Multiple studies have focused on the AD markers amyloid-β 42 (Aβ_42_), total tau (t-tau), phosphorylated tau (p-tau), and α-Syn. Recent studies have shown that low CSF Aβ_42_ levels were related not only to cognitive impairment in PD but also to rapid cognitive decline (Bäckström et al., 2015; Stav et al., 2015). T-tau, p-tau, and α-Syn levels at each disease stage are inconsistent. CSF t-tau, p-tau, and total α-Syn levels may increase with disease stage, and high levels of these factors have been associated with cognitive impairment in patients with more advanced disease, but not those at an early disease stage (Hall et al., 2016). The above results suggest that CSF Aβ_42_, t-tau, p-tau, and total α-Syn can be used as objective indicators of cognitive function in PD experimental models.

The diagnosis of most diseases mainly depends on the detection of biomarkers in the serum or plasma. Glucosylceramidase (GBA), leucine-rich repeat serine/threonine-protein kinase 2 (LRRK2), and α-Syn mutations have been associated with PD-MCI and PDD (Aarsland et al., 2017). Recent studies have shown that high levels of serum/plasma α-Syn and low levels of serum glial cell line-derived neurotrophic factor (GDNF), miRNA-29s (miR-29s), vitamin D, and 25-hydroxyvitamin D may be involved in the cognitive impairment of PD-MCI and PDD patients (Bougea et al., 2020; Han L. et al., 2020; Liu Y. et al., 2020; Santangelo et al., 2020). Importantly, low levels of epidermal growth factor (EGF) may be predictive of cognitive decline (Pellecchia et al., 2013). However, the current body of literature on the association between serum/plasma biomarkers and cognitive impairment lacks long-term randomized controlled trials with multi-center large samples. Furthermore, the combination of CSF and serum markers was significantly better than serum alone at distinguishing between PDD and PD (Bougea et al., 2020). Although additional novel biomarkers have been found to be related to cognitive impairment in PD, Aβ_42_, t-tau, p-tau, and α-Syn are relatively reliable indicators of cognitive function.

### Neuroimaging

Over the past decade, our understanding of the association between morphologic changes and cognitive impairment in PD has been characterized using structural and molecular neuroimaging techniques, such as MRI and PET. Although PD-MCI might show evidence of cortical volume loss, PDD was associated with extensive cortical and subcortical atrophy that can be quantified with a structural MRI (Duncan et al., 2013). Patients with PD-MCI have been found to have cortical volume loss in the parietal, posterior, and frontal cortices and atrophy in the hippocampus (Mak et al., 2015; Pereira et al., 2015; Yildiz et al., 2015). In contrast, PDD has more severe cortical thinning in the parietal, occipital, temporal, and frontal cortices and substantial atrophy of the hippocampus, including the parahippocampus, insula, and cingulate gyrus (Aarsland et al., 2017).

Prior works have also focused on the association between cognitive impairment and PET on dopaminergic (DAergic) molecular, glucose metabolism, and cholinergic molecular interactions. Several studies have suggested that PD-MCI has DAergic deficits in the striatum and the insula, while such defects in the PDD are in the striatum, anterior cingulate, and midbrain (Chou et al., 2014; Aarsland et al., 2017; Politis et al., 2017; Han et al., 2021). It was reported that [C-11]-PE2I (N-(3-iodoprop-2E-enyl)-2b-carbomethoxy-3b-(4-methyl-phenyl)nortropane) (PE2I), a PET radiotracer targeting neuronal dopamine (DA) transporters with high specificity, avidity in the caudate, putamen, and pallidum was indicative of more severe cognitive function deficits (Ivanidze et al., 2020). Moreover, PET imaging of glucose metabolism has shown lower levels of metabolic activity in the parietal, temporal, cingulate, and frontal cortices of patients with PD-MCI and PDD (González-Redondo et al., 2014; Ruppert et al., 2021). The cholinergic system, which is responsible for cognitive processing and is associated with the clinical manifestations of PDD, plays an important role in the structural and functional remodeling of the cortical circuits (Aarsland et al., 2017). PDD has been associated with the loss of cholinergic function in the temporal, frontal, and medial occipital cortices and the thalamus (Kotagal et al., 2012; Aarsland et al., 2017). However, most studies only identified a relationship between PET and cognitive impairment in patients with PDD, not PD-MCI.

## Systematic Literature Search on Experimental Models of Parkinson’s Disease Cognitive Impairment

**FIGURE 1:**
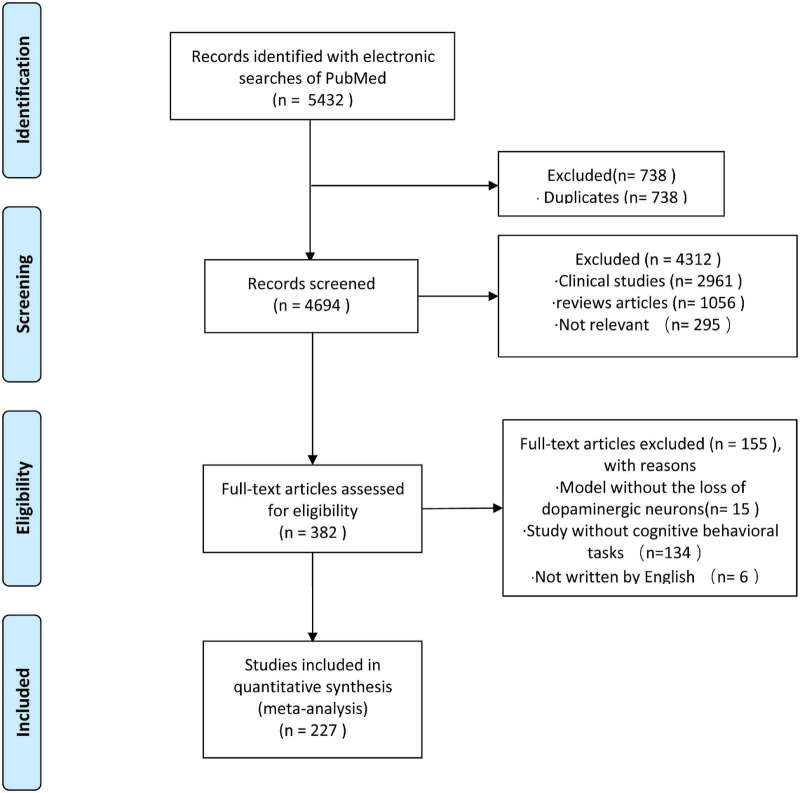
Flow chart for the selection of studies.

To clarify trends in experimental modeling of PD cognitive impairment, we systematically searched PubMed using the following search terms: (“Parkinson’s disease [Title/Abstract]” AND “cognitive function [Title/Abstract]”) OR (“Parkinson’s disease [Title/Abstract]” AND “cognitive impairment [Title/Abstract]”) OR (“Parkinson’s disease mild cognitive impairment” AND “model”) OR (“Parkinson’s disease dementia” AND “model”). The publication range was from the start of the database to 30 May 2021. Two investigators (YF and JH) reviewed each topic and abstract independently to select references that met the inclusion criteria. Inconsistencies encountered by the reviewers would be resolved after consultation with a third reviewer (DC). The selection process is shown in Figure 1. We identified 5432 articles, of which 738 were duplicates. A total of 227 articles met our inclusion criteria and were included in the analysis.

**FIGURE 2:**
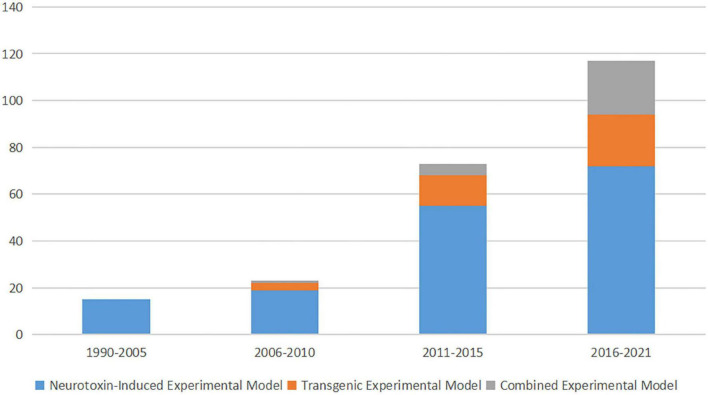
Trends in experimental models of PD with cognitive impairment.

Information on the experimental models used in each work was extracted. Models were divided into three categories based on model generation: neurotoxin-induced experimental model, transgenic experimental model, and combined experimental model. Models using any kind of neurotoxic substance were defined as a neurotoxin-induced experimental model. The transgenic experimental model group included models that involved gene manipulation. Models that included two or more methods were assigned to the combined experimental model group. Figure 2 demonstrates an increased focus on cognitive impairment in PD over time. Although neurotoxin-induced experimental models were the most prevalent during every time period, transgenic experimental models and combined experimental models have received increased recent attention.

## Neurotoxin-Induced Experimental Models of Cognitive Impairment

### 1-Methyl-4-Phenyl-1,2,3,6-Tetrahydropyridine-Induced Experimental Models

1-Methyl-4-phenyl-1,2,3,6-tetrahydropyridine has been commonly used in experimental models of PD. MPTP is a highly lipophilic prodrug that crosses the blood–brain barrier (BBB) into the brain, where it is metabolized into the potent neurotoxin 1-methyl-4-phenylpyridinium (MPP+). MPP+ released from astrocytes into the extracellular space is taken up by neighboring DAergic neurons. It consequently becomes highly concentrated in the microglia and induces oxidative stress and decreased adenosine triphosphate (ATP) generation by inhibiting mitochondrial complex I of the mitochondrial electron transport chain (El-Gamal et al., 2021). This leads to apoptosis and necrosis of DAergic neurons due to the rapid decrease in the concentration of ATP in the SN and striatum.

A total of 73 articles showed that MPTP therapy alone can induce both the loss of DAergic neurons in the SN and clinical cognitive deficits (Table 2). An initial MPTP-treated PD model with cognitive deficits in macaque nemistrina monkeys was reported in 1990 (Schneider and Kovelowski, 1990). Based on different experimental conditions and requirements, scholars have created 16 modeling methods for MPTP-induced PD cognitive impairment using seven different methods of drug administration: oral (p.o.), intravenous (i.v.), intramuscular (i.m.), subcutaneous (s.c.), intraperitoneal (i.p.), stereotaxical, and intranasal (pr.nar.) (Table 2). Rodent and non-human primate (NHP) models have primarily been used to model PD with cognitive impairment. This suggests that MPTP is easily absorbed by animals and that experimenters require personal protection.

**TABLE 2:** Methods of MPTP-induced experimental models.

Experimental subject			Method			Motor symptoms of Parkinson’s disease	Cognitive behavioral test	Type of cognitive impairment	Mechanism of action	Limitations
Strain		Age	Injection	Dose	Mold time					
Rodent	• C57/BL6N mice • C57B/6JRj mice • C57BL/6 mice • C57BL/6J mice • C57BL/6N mice • Swiss albino mice • SD rat • Wistar rat	28–180 days old	i.p.	• 20 mg/kg/2 h for 4 times • 10–30 mg/kg/day for 4–8 times • 25–30 mg/kg/3.5 days for 5–10 times	5–35 days	• Tremor, muscle rigidity, hypotonia, hypokinesia, hollow back, catalepsy and dysfunctional dynamic gait • Loss of balance and motor coordination in the pole test, beam walk test and rotarod test • Transitory hypolocomotion	• NOR • MWM • PAT • Barnes maze test • T-maze test • Y maze test • SRT • SORT • Episodic-like memory test	• Deficits in short-term spatial learning, working memory, memory acquisition and retention, but not long-term • Short-term social and object recognition memory • Short-term inhibitory avoidance memory deficit • Contextual memory deficit • Visuospatial attention deficits • Episodic-like memory deficit	• Degeneration of nigrostriatal DAergic neurons • Striatal DA depletions • Abnormal accumulation of α-Syn • Microglial activation in the SN, striatum, and hippocampus • Cell loss in the hippocampal CA1 and DG area • Declines in the DA transporter in the medial and lateral caudate, ventral and dorsal putamen, especially D3Rs • High level of the enzyme proline endopeptidase • Neuroinflammation • Autophagy pathway • Apoptotic mechanisms • Hyperactivity of the glutamatergic system • Destroy the mtDNA and nDNA integrity	• High mortality in rodents • Neurological damage tends to heal spontaneously • Environmental exposure
		
			pr.nar.	• 1 mg/nostril for 14 days • Totaled 65 mg/kg • 1 mg/nostril/day for 1 time • 0.1 mg/nostril/day for 21 days	7–21 days					
		
			s.c.	• 25 mg/kg/3.5 days for 10 times	35 days					
		
			Stereotaxical injection (SN)	100 or 209.72 μg/side	7–28 days					
	
NHP	• *Cynomolgus macaque* monkey • *Macaca fascicularis* monkey • *Macaca mulatta* monkey • *Macaca nemistrina* monkey • *Marmoset* • *Rhesus* monkey • *African green* monkey; • *Squirrel* monkey	2–15 years old	i.v.	• Beginning at 0.01 or 0.05 mg/kg and increasing up to 0.175 or 0.25 mg/kg, 2–3 times/week for 5–13 months • 0.025–0.5 mg/kg/time, 2–3 times/week for 27–450 days	27–390 days	• Tremors, bradykinesia, and abnormal posture and decreased activity, low frequency (4.3 ± 1.7 Hz) tremor of the head at rest • High motor symptom score	• VDR • CPT • MDR • SDR • ORDT • DM-ST • DALT • ASST • VPDT	• Spatial working memory deficit • Attention deficit • Learning deficit • Executive function deficit • Deficit in modulate prefrontal cortex-mediated cognitive performance		
		
			i.m.	• 0.05–0.4 mg/kg, 2 time/week for 12–72 times • 0.1 mg/kg/4–5 days for 58 weeks, then 0.4 mg/kg/week for 4–24 weeks	42–574 days					
		
			s.c.	• 0.2–2 mg/kg/day for 3–5 times • 2 mg/kg for 1st time, 1.9 mg/kg for 2nd time an interval of 3–4 weeks	3–28 days					
		
			p.o.	0.05–0.15 mg/kg, 2–3 times/week for 4 months	120 days					

*NHP, non-human primates; SD rat, Sprague Dawley rat; p.o., oral administration; i.v., intravenous injection; i.m., intramuscular injection; s.c., subcutaneous injection, i.p., intraperitoneal injection; pr.nar., intranasal injection; NOR, novel object recognition test; MWM, Morris water maze task; PAT, passive avoidance test; SRT, Social recognition test; SORT, social odor recognition task; VDR, variable delayed response task; CPT, continuous performance task; MDR, modified delayed response task; SDR, spatial delayed response task; ASST, attentional set-shifting test; ORDT, Object Retrieval Detour Task; DM-ST, Delayed Matching to-Sample testing; DALT, delayed alternation test; VPDT, Visual Pattern Discrimination task; DAergic, dopaminergic; DA, dopamine; α-Syn, α-synuclein; D3Rs, dopamine D3 receptor; SN, substantia nigra; DG, dentate gyrus.*

From 1990 to 2010, the majority of MPTP-treated experimental models used NHP to study cognitive impairment, including *Cynomolgus macaque*, *Macaca fasciculari*, *Macaca mulatta*, and eight other kinds of monkeys, on account of the specific sensitive and reproducible toxic effects of MPTP on the DAergic nigrostriatal pathway of NHPs (Taylor et al., 1990; Schneider and Pope-Coleman, 1995; Decamp and Schneider, 2009; Schneider et al., 2013). VDR, CPT, modified delayed response task (MDR), spatial delayed response task (SDR), attentional set-shifting test (ASST), and nine other kinds of tasks are commonly used to monitor the cognitive deficits of primates (Fernández-Ruiz et al., 1999; Millan et al., 2010; Schneider et al., 2013; Ko et al., 2016). Previous studies demonstrated that MPTP-treated NHPs developed motor dysfunction similar to that seen in PD, including tremors, bradykinesia, abnormal posture, decreased activity, low frequency (4.3 ± 1.7 Hz) tremor of the head at rest, and a high motor symptom score (Zhang et al., 2013; Choudhury and Daadi, 2018). NHP models also demonstrated evidence of cognitive impairments, such as spatial working memory deficits, attention deficits, learning deficits, executive function deficits, or deficits in the modulation of PFC-mediated cognitive performance (Schneider et al., 2002, 2013; Zhang et al., 2014; Ko et al., 2016; Phillips et al., 2017). A slowly progressing dose of MPTP administered to monkeys (ranging from an initial 0.05 mg/kg to 0.075 mg/kg, 2 to 3 times per week for up to 24 weeks) resulted in the development of cognitive deficits before motor deficits (Schneider and Pope-Coleman, 1995), which suggested that an MPTP-treated monkey model can yield significant individual differences in motor and cognitive impairment. There are several drawbacks to NHP models that limit their use in the study of mechanisms of cognitive impairment. First, the Unified Parkinson’s Disease Rating Scale, which assesses the severity of the disease phenotype, has not been validated in NHPs (Imbert et al., 2000). Secondly, a long experimental period, high costs, a complicated genetic manipulation process, and ethical considerations limit the employment of NHP in research (Chia et al., 2020).

As NHP studies require a high experimental threshold, rodents are valuable experimental subjects when evaluating cognitive impairment in PD. The motor symptoms of PD can also be induced in rodent models by MPTP, including tremors, muscle rigidity, hypotonia, hypokinesia, hollow back, catalepsy, dysfunctional dynamic gait, transitory hypolocomotion, loss of balance, and poor motor coordination. Cognitive impairment phenotypes in rodents can be quantified using a series of tests, such as the MWM, NOR, passive avoidance test (PAT), Barnes maze test, T-maze test, and Y-maze test. However, one report noted that acute (4 mg/kg/day, 4 days, i.p.) and chronic (22 mg/kg/day, 28 days, i.p.) MPTP-treated models did not develop long-term changes in locomotive and cognitive function (Fifel et al., 2013). Most studies have suggested that an MPTP-treated rodent model induces specific types of cognitive impairment, which include deficits in short-term spatial learning, working memory, memory acquisition and retention, short-term social and object recognition memory, short-term inhibitory avoidance memory, contextual memory, visuospatial attention, and episodic-like memory (Monaghan et al., 2010; Wang et al., 2010; Castro et al., 2012; Das et al., 2014; Ho et al., 2014; Yabuki et al., 2014; Zhang et al., 2018; Yang et al., 2020).

The above studies suggest that cognitive deficits in rodents are linked to degeneration of the nigrostriatal DAergic neurons, striatal DA depletions, and neuroinflammation (Perry et al., 2004; Wang et al., 2009; Hsieh et al., 2012a). In addition, α-Syn and phosphorylated-α-synuclein (p-α-Syn), which are significant contributors to the neuropathology of PD-MCI and PDD, accumulated abnormally in the striatum and SN of MPTP-treated mice (Han N. R. et al., 2020). Moreover, microglial activation in the SN, striatum, and hippocampus and cell loss in the hippocampal CA1 and dentate gyrus (DG) areas are related to deficits in the cognitive function of MPTP-treated rodents (Ho et al., 2011; Hsieh et al., 2012b). Due to differences in protocol and animal strains, reports to this end have presented inconsistent findings. However, these experimental models have been able to reproduce the partial cognitive impairment observed in parkinsonian patients. Several limitations of MPTP-treated experimental models cannot be ignored, which include the high mortality rate of treated rodents, neurologic damage that tends to heal spontaneously, and environmental exposure effects.

### 6-Hydroxy Dopamine-Induced Experimental Models

Treatment using 6-OHDA, a selective catecholaminergic neurotoxin that cannot cross the BBB, leads to a consistent behavioral phenotype in experimental models and predictable degeneration of DAergic neurons. In the 62 articles that utilized a 6-OHDA-treated experimental model, multiple different types of cognitive impairment in addition to motor symptoms were induced (Table 3). Except for zebrafish, which were exposed to 6-OHDA by dissolving it into the culture medium, rodent and gray treefrog models received stereotactic injections of 6-OHDA into different anatomic regions (Endepols et al., 2004; Ramos-Moreno et al., 2012; Choi et al., 2019; Abidar et al., 2020) such as the SN, the MFB, the striatum, PFC, VTA, hippocampus, and the third ventricle (Gasbarri et al., 1996; Gu et al., 2012; Kadowaki Horita et al., 2013; Razavinasab et al., 2013; Zhou et al., 2017; Bayo-Olugbami et al., 2020; Schneider et al., 2021). Of these, the target proportions for unilateral stereotactic 6-OHDA injection were more common in the MFB and the SN, whereas the target proportions for bilateral injection were more common in the striatum. Previous studies showed that the injection of 6-OHDA into the SN or the MFB led to rapid and enormous DA cell body degeneration, which involves the nigrostriatal pathway in an anterograde progression. Moreover, the administration of 6-OHDA into the striatum affected DA terminals and disrupted the nigrostriatal pathway in retrograde succession (More et al., 2016). Although there was a wide total dose range of 6-OHDA used in the literature (3–200 μg), 8–24 μg was most commonly used (Hsueh et al., 2018; Wang et al., 2019).

**TABLE 3:** Methods of 6-OHDA-induced experimental models.

Experimental subject			Method			Motor symptoms of Parkinson’s disease	Cognitive behavioral test		Type of cognitive impairment	Mechanism of action	Limitations
		
Strain		Age	The target proportion	Total dose	Mold time						
Rodent	• C57/BL6 mice • C57B/6J mice • C57BL/6 mice • C57BL/6J mice • C57BL/6N mice • CD1 mice • SD rat • Wistar rat • Lister Hooded rat • Long Evans rat	42–600 days old	• MFB • SN • Striatum • PFC • VTA • Hippocampus • The third ventricle	3–200 μg	7–140 days	• Specific stereotyped rotation after subcutaneous injection of apomorphine (unilateral injection) • Hypolocomotion • Shuffling gait as PD • Limb rigidity • Forepaw akinesia in limb-use asymmetry test • Loss balance and coordination in rotarod test	• MWM • NPR • CFBT • DAT • SRT • VDT	• NOR • PAT • SAT • SDAT • TVOCRT • 5-CSRT	• Long-term spatial learning acquisition and memory retention deficits, but not short-term • Spatial and object location memory deficits • Long-term object recognition deficit • Visuo-spatial memory deficit • Deficits in long- and short-term working memory and provisionally on additional executive processes • Cognitive flexibility performance deficits • Attentional deficit • Episodic-like memory deficit • Short-term social memory deficit	• Oxidative stress, neuroinflammation and DAergic neural apoptosis in SN, VTA, PFC, striatum and hippocampus • Mitochondrial dysfunction • Glial activation • Reduction of the levels of D3Rs and TH in the striatum • Reduction the levels of DA, DOPAC and HVA by reducing DA metabolism and transport • Increase of the expression of Aβ_1–42_, NOX4 in the DG of the hippocampus by activating MKK7-ERK-Fos-APP axis • Abnormal synaptic plasticity in the DG	• No formation of LBs • Bilateral lesion is associated with high mortality • Damage of the brain structure by stereotaxic injection • Lack of blood brain barrier penetration • Inconspicuous movement disorders in some articles • Cognitive dysfunction showed instability
	
							• Barnes maze test • Y-maze test • T-maze test • S&E task				
	
Zebrafish		90–120 days old	250 μM in culture medium		1 day	Unconspicuous	Y-maze test		Spatial memory deficit		
	
Gray treefrog		/	Telencephalic ventricles	6 μg	5 days	Phonotactic behavior impairment	Two-speaker experiments		Auditory memory deficits		

*SD rat, Sprague Dawley rat; SN, substantia nigra; MFB, medial forebrain bundle; PFC, the prefrontal cortex; VTA, the ventral tegmental area; NOR, novel object recognition test; MWM, Morris water maze task; PAT, passive avoidance test; SRT, social recognition test; NPR, Novel Place Recognition test; CFBT, cognitive flexibility behavioral tasks; SAT, sustained attention task; DALT, delayed alternation test; SDAT, stimulus discrimination acquisition test; TVOCRT task, two versions of the operant choice reaction time task; VDT, visual discrimination test; 5-CSRT, five-choice serial reaction time task; S&E task, spatial and egocentric task; DAergic, dopaminergic; D3Rs, dopamine D3 receptor; DOPAC, 3,4-dihydroxyphenylacetic acid; HVA, homovanillic acid; TH, tyrosine hydroxylase; DG, dentate gyrus; Aβ_42_, amyloid-β 42; LBs, Lewy bodies.*

The common motor symptoms seen in a 6-OHDA-treated rodent model include hypolocomotion, a shuffling gait similar to that seen in PD, limb rigidity, forepaw akinesia in the limb-use asymmetry test, and impaired balance and coordination in the rotarod test (Hadadianpour et al., 2017; Hsueh et al., 2018; Bayo-Olugbami et al., 2020). This model had a specific stereotyped rotation after subcutaneous injection of apomorphine if the 6-OHDA is injected unilaterally. These models also showed various levels of cognitive impairment as assessed with the MWM, NOR, five-choice serial reaction time task (5-CSRT), and the spatial and egocentric task (S&E task). These generally cover long-term spatial learning acquisition and memory retention deficits (but not short-term), spatial and object location memory deficits, long-term object recognition deficits, visuo-spatial memory deficits, deficits in long- and short-term working memory, and provisionally on additional executive processes, cognitive flexibility performance deficits, attention deficits, episodic-like memory deficits, and short-term social memory deficits (Tadaiesky et al., 2010; Hsieh et al., 2012b; Razavinasab et al., 2013; Eagle et al., 2015; Betancourt et al., 2017; Haghparast et al., 2018; Marshall et al., 2019). However, in cognitive studies, bilateral injection of a low dose of 6-OHDA (4–6 μg/side) is preferred as it produces a balanced loss of DAergic neurons and mimics the early pathologic changes of PD with cognitive impairment (Ferro et al., 2005).

In general, 6-OHDA induces rapid non-enzymatic auto-oxidation in the cytosol to produce toxic species, such as hydrogen peroxide, quinones, superoxide radicals, and hydroxyl radicals. These species can not only induce oxidative stress *via* the generation of ROS but also cause mitochondrial dysfunction by impairing mitochondrial complex I and inhibiting the activity of the electron transport chain (More et al., 2016). Previous studies have found that oxidative stress, neuroinflammation, and DAergic neural apoptosis in the SN, VTA, PFC, striatum, and hippocampus are related to the cognitive impairment of 6-OHDA-treated models, as the DAergic neurons present in the VTA, striatum and SN project their axons into the hippocampus and PFC (Bitu Pinto et al., 2015; Haghparast et al., 2018; Singh et al., 2018). Mitochondrial dysfunction and glial activation have also been shown to be involved in the cognitive impairment seen in 6-OHDA-treated models (Smith et al., 2017; Real et al., 2019). Two factors play an important role in 6-OHDA-treated models: reduced DA, 3,4-dihydroxyphenylacetic acid (DOPAC), homovanillic acid (HVA), dopamine D3 receptor (D3Rs) and tyrosine hydroxylase (TH) levels in the striatum, and reduced DA metabolism and transport (Goes et al., 2014; Betancourt et al., 2017). 6-OHDA injection increased the expression of Aβ_1–42_ and NOX4 in the DG of the hippocampus by activating the MKK7-ERK-Fos-APP axis in the PD-MCI model (Ba et al., 2019; Choi et al., 2019). Although 6-OHDA-treated experimental models serve as a useful tool for exploring the potential mechanism and therapies of PD-MCI and PDD, several significant disadvantages of this model need to be noted. Firstly, 6-OHDA fails to produce LBs, a key part of PD-MCI and PDD pathology. Further, bilateral lesions are associated with a high rate of mortality. Due to its inability to penetrate the BBB, stereotactic injection of 6-OHDA damages the structure of the brain. Moreover, several articles suggest that 6-OHDA-treated models generate inconspicuous movement disorders and unstable cognitive dysfunction (Tadaiesky et al., 2008; Carvalho et al., 2013; Oliveira et al., 2020; Schneider et al., 2021). Finally, 6-OHDA has to be injected together with noradrenaline and serotonin transport inhibitors because it is taken up by catecholaminergic nerve terminals.

### Novel Toxin-Induced Experimental Models

While MPTP and 6-OHDA are classic experimental inducers of PD in pre-clinical models, several pesticides, pharmacologic compounds and oligomers have been used to induce PD-like conditions that include DA depletion and cognitive deficits. A total of 26 articles describe 12 of these toxins (Table 4).

**TABLE 4:** Methods of novel toxin-induced experimental models.

Method				Experimental subject		Motor symptoms of Parkinson’s disease	Cognitive behavioral test	Type of cognitive impairment	Mechanism of action
		
Drug	Injection	Dose	Mold time (day)	Strain	Age (days old)				
Rotenone	500 μM in culture medium		7	Drosophila	8–10	• Loss motor coordination • Shuffling gait as PD	• T-maze APSA • Short-term memory assessment	Learning and short-term memory deficits	
	
	s.c.	2 mg/kg/day	21	SD rat	35–49	• Loss motor coordination in the rotarod test • Loss muscle strength • Reduction in the locomotion frequency	• Y-maze test • Barnes maze test • NOR • OLT • TWAAT	• Hippocampal-dependent spatial learning and memory deficits • Object recognition memory deficit	• DAergic neurons degeneration and apoptosis; • Neuroinflammation • Mitochondrial complex I inhibition
		
	Stereotaxical injection	5.4 μg (striatum)	7–93	• C57BL/6J mice • C57BL/6NCrl mice • SD rat • Wistar rat					
		12 μg/side (SNpc)							

Permethrin	p.o.	34 mg/kg/day for 16 days	29 or 179	• Wistar rat • Wistar rat pup	6 or 21	• Loss motor coordination • Shuffling gait as PD	• T-maze test • MWM	• Deficits in working memory, but not spatial memory	• Loss of DAergic neurons in SN • Reduction in DA and 5-HT levels

Reserpine	s.c.	• 0.1 mg/kg/2 days for 4, 10 or 20 times • 0.1 mg/kg/day for 3 weeks	8–40	• Swiss mice • Wistar rat	56–210	• Progressive catalepsy behavior and duration of oral twitching • Loss motor coordination	• NOR • Plus-maze discriminative avoidance task • MWM	• Long- and short-term memory and learning deficits • Short-term memory recognition deficit	• Degeneration of DAergic neurons in SN, VTA, GD, CA1, PFC and DS • Reduction in 5-HT and DA metabolism • An early effect on axonal ultrastructure

Ibotenic acid	Stereotaxical injection (STN; PPT)	10–18.9 μg/side	8 or 28	• SD rat • Wistar rat	250–330	Hypolocomotion	• Social interaction task • NOR	• Social and object recognition memory deficit	• Loss cholinergic tone • Disordered DAergic neurotransmission • Loss spinogenesis in hippocampus and PFC

Chlorpromazine	i.p.	3 mg/kg/day	21	Wistar rat	42–56	Loss grip strength	• MWM • PAT	Deficit in spatial learning and memory	Unclear

Haloperidol	i.p.	5 mg/kg/day	7	BLAB/c mice	30	Loss motor coordination	NOR	Deficits in recognition memory	Calcium mediated toxicity

H-αSynO	Stereotaxic injection (SNpc)	2.5 μg	150	SD rat	90	Loss motor coordination	NOR	Object recognition memory deficit	• Early mitochondrial loss and abnormalities in SNpc neurons
Recombinant α-synuclein fibrils	Stereotaxic injection (striatum)	300 μM/side	180	C57BL/6J mice	80	Unconspicuous	Cued and contextual fear conditioning tube test	Deficits in fear memory and social dominance	Impairment in cortical and amygdala function, without causing cell loss

LPS	Stereotaxic injection (SNpc)	• 10 μg/side • 0.8 μg/time for 4 times, 4 days interval between each infusion	14 or 30	Wistar rat	90	Deficits in grip strength, motor balance and coordination	• MWM • NOR	• Spatial learning and memory deficit • Recognition memory deficit	Oxidative damage and neuroinflammation on the DAergic system

MnCl_2_	i.p.	20 mg/kg	62	Wistar rat pup	8	Unconspicuous	• NOR • SRT	Deficits in short-term memory and recognition memory	Unclear

BMAA	s.c.	400 mg/kg/day on postnatal day 3, 4, 5, 6 or 7	108	SD rat pup	3	• Catalepsy behavior; • Shuffling gait as PD	• Barnes maze test • LTRMT • MWM	Deficits in long-term working and reference memory, short-term memory and learning, spatial navigation and reference memory	BMAA is selectively toxic to the hippocampal neuronal population, alterations in the levels of DA and/or serotonin

BSSG	p.o.	3 mg/day, 5 days a week for 4 months	300	SD rat	90	Methamphetamine triggered a rotational response	• Barnes maze test • SA-T-maze test	Deficits in short-term working memory, reference and spatial memory	Progressive loss of nigral DArgic neurons, a progressive spread of α-Syn pathology

*SD rat, Sprague Dawley rat; s.c., subcutaneous injection, i.p., intraperitoneal injection; p.o., oral administration; STN, subthalamic nucleus; PPT, pedunculopontine tegmental nucleus; SN, substantia nigra; PFC, the prefrontal cortex; VTA, the ventral tegmental area; T-maze APSA, T-maze Aversive Phototaxis Suppression Assay; NOR, novel object recognition test; MWM, Morris water maze task; PAT, passive avoidance test; SA-T-maze test, spontaneous alternation T-maze test; SRT, social recognition test; LTRMT, long-term reference memory test; DAergic, dopaminergic; DG, dentate gyrus; DS, dorsal striatum; LPS, lipopolysaccharide; BSSG, β-sitosterol β-d-glucoside; BMAA, β-N-methylamino-L-alanine; DA, dopamine; α-Syn, α-synuclein.*

Because pesticide exposure is a well-known risk factor of PD, rotenone and permethrin are useful tools for simulating environmental exposure. Rotenone, an inhibitor of mitochondrial complex I by penetrating the BBB in a manner similar to MPTP, has been shown to induce PD-like motor symptoms and learning and memory deficits in rodents and drosophila (Dhanraj et al., 2018; Perez-Pardo et al., 2018; Bandookwala et al., 2019). Interestingly, rapid eye movement sleep deprivation, which is a way to damage cognitive function, improved memory impairment in a rotenone-treated rat model due to moderate compensatory striatal DA release (Dos Santos et al., 2013). A previous study found that LBs can be formed in the surviving DAergic neurons in a rotenone model; however, this has not been observed in models of cognitive impairment (Inden et al., 2011). Rats exposed to permethrin early in life can also develop a phenotype of PD that is characterized by poor motor coordination, gait changes, and working memory deficits due to oxidative stress, LB formation, mitochondrial complex I defects, and DA loss (Nasuti et al., 2013, 2017). While cognitive impairment observed in both rotenone- and permethrin-treated models appears to be related to DAergic neuron degeneration, the exact mechanism that causes this effect remains unclear.

Several pharmacological compounds, including reserpine, ibotenic acid, chlorpromazine, and haloperidol, have been used to induce reversible DA depletion (Camacho-Abrego et al., 2014; Ogundele et al., 2015; Campêlo et al., 2017; Naeem et al., 2017). Reserpine exposure decreases brain monoamine storage capacity and total volume by inhibiting the vesicular monoamine transporter VMAT2, which models early-stage PD that progresses to PDD (Ikram and Haleem, 2019; Leal et al., 2019). Reserpine-treated rodent models showed not only progressive catalepsy behavior and periods of oral twitching and reduced motor coordination but also developed impaired long- and short-term memory and learning (Campêlo et al., 2017; Leal et al., 2019). Stereotactic injection of ibotenic acid into the subthalamic nucleus (STN) and pedunculopontine tegmental nucleus (PPT) can affect cholinergic neurons and the cholinergic system, thereby disturbing DAergic neurotransmission, which is associated with learning and memory (Targa et al., 2018). Although chlorpromazine and haloperidol may trigger PD cognitive impairment, this has not been well demonstrated and requires further validation (Ogundele et al., 2015; Naeem et al., 2017).

The novel toxins human α-synuclein oligomers (H-αSynO) and recombinant α-synuclein fibrils can induce PD memory deficits by depleting early mitochondria in SN neurons, which damages nigrostriatal DAergic neurons and inhibits cortical and amygdala function (Boi et al., 2020; Stoyka et al., 2020). These toxins can also induce p-α-Syn deposition in the SN neurons and microglia and spread to the striatum, which is a similar pathology to that of PD-MCI and PDD (Boi et al., 2020).

Lipopolysaccharide (LPS), an endotoxin, has been used to simulate PD neuroinflammation, which can induce spatial learning and memory deficits and recognition memory deficits (Delattre et al., 2013; Saini et al., 2020). Although other novel toxins, including MnCl_2_, β-sitosterol β-d-glucoside (BSSG), β-*N*-methylamino-L-alanine (BMAA), have been found to trigger PD with cognitive deficits, their reliability still requires further verification (Peres et al., 2015; Van Kampen et al., 2015; Scott and Downing, 2017).

## Transgenic Experimental Models

Only 5%–15% of PD cases are attributed to clear genetic factors; however, transgenic experimental models play a significant role in the study of idiopathic PD-MCI and PDD. In addition to common familial PD-linked genes, which include α-Syn, parkin, PTEN-induced putative (PINK1), protein deglycase (DJ-1), and LRRK2, 11 other transgenic models were used in a total of 38 articles to replicate PD-MCI or PDD (Table 5). The genes that these models interfered with included Pitx3, dopamine transporter (DAT), mitochondrial transcription factor A (TFAM), β-synuclein (β-Syn), A2A receptors, Atp13a2, B4galnt, Ifnb, midkine (Mdk), Ndufs4, tau, and Washc4 (Prediger et al., 2011; Lei et al., 2012; Sekiyama et al., 2012; Li et al., 2013; Moon et al., 2013; Schultheis et al., 2013; Ejlerskov et al., 2015; Choi et al., 2017; Hwang et al., 2019; Wu et al., 2020; Courtland et al., 2021). Rodent, NHP, and drosophila animal models based on α-Syn are currently available. Rodents and drosophila have been used in LRRK2 transgenic models. However, other rodent transgenic models have also been established.

**TABLE 5:** Methods of transgenic experimental models.

Transgenic animal				Motor symptoms of Parkinson’s disease	Cognitive behavioral test		Type of Cognitive Impairment	Mechanism of Action
Gene	Strain	Transgenic method	Age (cognitive impairment appears)					
α-Syn	Rodent	Mutation	4–12 months old	• Freezing behavior • Shuffling gait as PD • Loss motor coordination in the nesting task, rotarod test, pole test and hindlimb clasping behavior test	• Y-maze test • T-maze test • Barnes maze test	• PAT • TCSIT • MWM	• Object recognition memory • Deficits in long-term and short-term spatial learning and working memory • Social memory deficit	• Tau oligomers were elevated in hippocampus, pons and cerebellum • Reduction in dendritic spine density of hippocampal and caudate putamen medium spiny neurons • Lower synaptophysin and synapsin I levels in the frontal cortex and hippocampus • microglial response
		
					• Operant learning task			
		Knockout	2 months old	Unconspicuous	• PAT • AAT • MWM		Deficits in long-term learning and spatial memory	α-Syn is necessary for long-term spatial and working memory in hippocampus and limbic system
		
	NHP	Mutation	30–36 months old	Stereotypic behaviors that were repetitive, unvarying actions without goal or function	CPT		Deficits in learning and memory	Unclear
	
	Drosophila	Mutation	unclear	Unconspicuous	Courtship assay		Reduction of courtship behavior	Human α-Syn and Lewy bodies specifically damage the DAergic neurons, leading to the low content of the DA

LRRK2	Rodent	Mutation	8.6–21 months old	Loss motor coordination in rotarod test	• T maze test • Barnes maze test • Novel arm discrimination test		• Spatial recognition memory deficit • Unconspicuous working memory deficit	• Late-stage DA transmission deficits in the dorsal striatum • Low expression of PSD-95 in hippocampus
	Drosophila	Mutation	0.1 months old	Unconspicuous	• Classical olfactory conditioning in a T-maze		Deficit in long-term memory but not the short-term memory	• Decreasing the calcium channel activity of KCs

PINK	Rodent	Knockout	4 months old	Unconspicuous	• NOR • Barnes maze test		• Deficit in recognition memory • Spatial learning and memory deficit	• Low levels of glutathione, ATP, and elevated oxidative stress in the SN, striatum and deep cerebellar nuclei

DJ-1	Rodent	Knockout	10 months old	Loss motor coordination in rotarod test	MWM		Deficits in spatial learning and memory	DJ-1 can render neurons more resistant to oxidative stress and to misfolded protein accumulation

Parkin	Rodent	Knockout	5–6 months old	Unconspicuous	• OLT • Modified Y-maze test		Deficits in short-term spatial memory	• Deficiencies in hippocampal synaptic plasticity • [3H]DA release is increased in striatal synaptosomes

Pitx3	Rodent	Knockout	2–3 months old	• Lumbering behavior • Loss motor coordination in the pole test	• PAT • Inhibitory avoidance test • Swimming and dry T-maze	Deficits in learning and memory	The robust loss of midbrain DA neurons

DAT & TFAM	Rodent	Knockout	2–5 months old	• Progressive loss of motor function • Loss motor coordination	• Barnes maze test • NOR • MWM	Deficits in spatial learning and memory and cognitive function	Specific inactivation of TFAM in DAergic neurons

β-Syn	Rodent	Overexpress	3 months old	Unconspicuous	MWM	Deficit in spatial learning and memory	Protein aggregation and astrogliosis

A2A receptors	Rodent	Overexpress	Unclear	Unconspicuous	• 6-arm radial tunnel maze test • T-maze test • NOR • MWM	Deficits in working memory, certain spatial long-term reference memory, slower learning process	Increased A2A mRNA levels and A2A receptor protein levels as well as of increased A2A receptor binding function especially in regions of the cerebral cortex

Atp13a2	Rodent	Knockout	20–29 months old	Shuffling gait as PD	• NOR • MWM	Deficit in recognition of a novel object	Loss of Atp13a2 causes a-Syn accumulation and accumulation of lipofuscin deposits characteristic of NCL

B4galnt	Rodent	Knockout	7–10 months old	Low muscle strength	T maze forced-trial spontaneous alternation test	Deficit in short-term spatial memory	Partial deficiency of the GM1 family of gangliosides

Ifnb	Rodent	Knockout	3 months old	Motor coordination, balance, and grip strength deficit	MWM	Deficits in spatial learning and memory	Lack of neuronal IFN-β-IFNAR signaling causes brain Lewy body accumulation/IFN-β deficiency causes late-stage autophagy block

Mdk	Rodent	Knockout	3–4 months old	Unconspicuous	SRT	Deficit in short-term memory recognition	• Partial loss of DAergic neurons in the SNpc and depletion of DA and its metabolites in the olfactory bulb and striatum

Ndufs4	Rodent	Knockout	9 months old	Unconspicuous	• NOR • OLT • MWM	Deficits in short- and long-term memory, spatial learning and memory	Mitochondrial complex I dysfunction in DAergic neurons

tau	Rodent	Knockout	12 months old	Loss motor coordination in the pole test, rotarod test	Y-maze test	Deficit in working memory	Loss of soluble tau could contribute to toxic neuronal iron accumulation

Washc4	Rodent	Mutation	5.5 months old	Both gait and strength functions deteriorated.	• Y-maze test • NOR	Deficit in working memory, short- and long-term object recognition memory	Endo-lysosomal dysfunction in the brain

*α-Syn, α-synuclein; PINK1, TEN-induced putative; DJ-1, protein deglycase; LRRK2, leucine-rich repeat serine/threonine-protein kinase 2; DAT, dopamine transporter; TFAM, mitochondrial transcription factor A; β-Syn, β-synuclein; Mdk, midkine; NOR, novel object recognition test; MWM, Morris water maze task; PAT, passive avoidance test; TCSIT, Three-Chamber Social Interaction Test; AAT, Active Avoidance Task; CPT, continuous performance task; OLT, object location task; SRT, social recognition test; DAergic, dopaminergic; DA, dopamine; KCs, Kenyon cells; ATP, adenosine triphosphate.*

α-Synuclein, a small neuronal protein present at presynaptic terminals, regulates membrane and vesicular dynamics (Kin et al., 2019). Transgenic models of α-Syn, such as those with mutations (A53T and A30P) or gene knockout, have developed cognitive deficits similar to PD-MCI or PDD (Freichel et al., 2007; Kokhan et al., 2012; Singh et al., 2019). As tau oligomers are elevated by α-Syn in the hippocampus, pons, and cerebellum, A53T mice showed not only freezing behavior and loss of motor coordination but also long- and short-term spatial learning and memory deficits at 12 months (but not 6 months) of age (Singh et al., 2019). Several mechanisms are related to the motor and cognitive deficits seen in α-Syn transgenic models, such as reduced dendritic spine density of the hippocampal and caudate putamen medium spiny neurons, lower synaptophysin and synapsin I levels in the frontal cortex and hippocampus, and impaired microglial response (Table 5). Moreover, α-Syn transgenic NHP models of PD develop more replicable and stable phenotypes and pathologic changes compared with those of toxin-treated NHP models of PD (Niu et al., 2015). Interestingly, α-Syn knockout mice also developed long-term learning and spatial memory deficits, which suggests that appropriate α-Syn is necessary for cognitive function of the hippocampus and limbic systems (Kokhan et al., 2012). Therefore, human α-Syn A53T and A30P appear to play a key role in the mechanisms of PD-MCI and PDD.

R1441C/G and G2019S are the two most common LRRK2 mutations that are related to the pathologic mechanisms of the autosomal dominant inheritance of familial PD. LRRK2 mutation models may be most useful for studying early compensation in the nigrostriatal DA system because they demonstrate loss of motor coordination and spatial recognition memory deficits consistent with late-stage DA transmission deficits in the dorsal striatum and low expression of PSD-95 in the hippocampus (Sloan et al., 2016; Adeosun et al., 2017).

Gene knockout (KO) models, including parkin, PINK1, DJ-1, Pitx3, Mdk, and Ndufs4, are useful models of the earliest abnormalities in the nigrostriatal DA system during PD pathogenesis. MitoPark (DAT^+/cre^-Tfam^loxP/loxP^) mice and TGR (NSEhA2A) rats may play an important role in studies that involve the DA transporter. Transgenic models that more closely resemble human PD physiology and progression are valuable in the development of preventative treatments. However, it is not clear if results that are obtained from transgenic models can be applied directly to humans because most of the splicing events between humans and rodents are conserved (Kin et al., 2019). On the other hand, some types of transgenic models can serve as novel tools for generating different phenotypes of cognitive deficits and unstable motor symptoms of PD, although the repeatability and stability of these phenotypes require further verification. Thus, we should objectively analyze the results of transgenic models in future work, such as by observing the relationship between cognitive impairment and changes in different regions of the brain during transgenic animal development and aging using advanced imaging.

## Combined Experimental Models

In order to imitate the complex pathogen and neuropathology of PD-MCI and PDD, combined experimental models have become increasingly popular. The number of articles that utilized models has gradually increased. A total of 24 kinds of combined experimental models were reported in 30 articles and can be divided into three categories: dual/multiple toxin-induced models, models combining toxins and transgenes, and models combining toxins and surgery (Tables 6–8).

**TABLE 6:** Methods of dual/multiple toxins induced models.

Method	Strain	Age (days old)	Mold time (day)	Motor symptoms of Parkinson’s disease	Cognitive behavioral test	Type of cognitive impairment
6-OHDA (10 μg, SN) + LPS (250 μg/kg/day, 7 times)	Rodent	35–42	14	Loss locomotor activity	• Y-maze test • Barnes maze test	Deficits in short-term, long-term spatial working memory
6-OHDA (12 μg/side, caudate putamen region) + saporin anti-orexin-B (10 ng/side, the LH/PeF)	Rodent	49	60	Unconspicuous	• NOR • MWM • T-maze test	Deficits in long-term memory and spatial memory, but not short-term memory and working memory
Combined paraquat (10 mg/kg) and maneb (30 mg/kg), 2 times/week, 12 times, i.p.	Rodent	42–90	42	Unconspicuous	• NOR • MWM	Deficits in recognition memory, spatial learning and memory
Combined DSP-4 (50 mg/kg), paraquat (10 mg/kg) and maneb (30 mg/kg), 2 times/week, 8 times, i.p.	Rodent	90	35	Unconspicuous	MWM	Deficit in spatial learning and memory
DSP-4 (25 mg/kg/day, 4 days, i.p.) + 6-OHDA (15 μg, striatum)	Rodent	56–64	25	Loss locomotor activity	NOR	Deficit in recognition memory
LPS (2.5 mg/kg/day, 3 times, i.p.) + recombinant monomeric human α-Syn (0.5 μM/site, striatum)	Rodent	56	30	Unconspicuous	• NOR • Y-maze test	Deficits in long-term recognition memory, short-term spatial memory

*6-OHDA, 6-hydroxy dopamine; LPS, lipopolysaccharide; DSP-4, N-(2-chloroethyl)-N-ethyl-2-bromobenzylamine; NOR, novel object recognition test; MWM, Morris water maze task.*

**TABLE 7:** Methods of models combining toxin and transgenes.

Method	Strain	Age (days old)	Mold Time (day)	Motor symptoms of Parkinson’s disease	Cognitive behavioral test	Type of cognitive impairment
6-OHDA (2.5 μg/side, striatum) + APP_swe_/PS1△E9 mice	Rodent	75–90	14	Loss balance and coordination in rotarod test	• Barnes maze test • Water T-maze	Deficit in hippocampus-dependent spatial and working memory
6-OHDA (2 μg/side, striatum) + CHT^HET^ mice	Rodent	77–91	18	Unconspicuous	• ASST • Spatial recognition test	Deficit in attention and long-term object recognition, but not in visual-spatial memory
MPTP (10 mg/kg/time, 10 times, i.p.) + mice expressing the human E4 isoform	Rodent	90–150	35	Unconspicuous	• MWM • NOR • Barnes maze test	Deficits in recognition memory, spatial learning and memory
MPTP (20 mg/kg/3 days, 7 times, i.p.) + hTau mice	Rodent	210–270	7	Gait instability	Barnes maze test	Deficit in long-term memory
MPTP (20 mg/kg/3 days, 7 times, i.p.) + TKO mice	Rodent	210–270	7	Gait instability	Barnes maze test	Deficit in long-term memory
MPTP (20 mg/kg/day, 3 times, i.p.) + nestin-GFP mice	Rodent	42	3	Loss motor coordination	MWM	Deficits in spatial learning and memory
Reserpine (0.3 mg/kg, 1 time, s.c.) + hD1 mice	Rodent	56–70	1	Loss locomotor activity	Y-maze test	Deficit in working memory
α-Syn oligomers (1 μM/side, striatum) + P*rnp*^0/0^ mice	Rodent	180–240	10	Unconspicuous	NOR	Deficit in recognition memory
α-Syn oligomers (1 μM/side, striatum) + TLR4^–/–^ mice	Rodent	56	10	Unconspicuous	NOR	Deficit in recognition, reversible memory
Human a-Syn brain extracts (55 ng/site, hippocampus) + 5xFAD Tg mice	Rodent	240	90	Unconspicuous	Y-maze test	Deficit in short-term working memory
AAV-α-Syn vector (0.5 × 10^9^ GC/μl/site, the medial prefrontal cortex) + human α-Syn preformed fibrils (2.5 μg/site, striatum)	Rodent	35–42	98	Unconspicuous	• DNMPT • 5-CSRT	Deficit in working memory, attention and inhibitory control
LPS (2.5 mg/kg/day, 3 times, i.p.) + A53T mice	Rodent	240	25	Gait instability	• NOR • Y-maze test • MWM	Deficits in long-term recognition memory, short-term spatial memory, learning and spatial memory

*6-OHDA, 6-hydroxy dopamine; MPTP, 1-methyl-4-phenyl-1,2,3,6-tetrahydropyridine; α-Syn, α-synuclein; LPS, lipopolysaccharide; NOR, novel object recognition test; MWM, Morris water maze task; ASST, attentional set-shifting test; DNMPT, the delayed non-matching to position test; 5-CSRT, five-choice serial reaction time task.*

**TABLE 8:** Methods of models combining toxin and surgery.

Method	Strain	Age (days old)	Mold Time (day)	Motor symptoms of Parkinson’s disease	Cognitive behavioral test	Type of Cognitive Impairment
Bilateral ovariectomy + 6-OHDA (250 μg/side or 8 μg/side, SN)	Rodent	• 35 • 150–180	• 14 • 35	Loss balance and coordination in rotarod test	MWM	Deficits in spatial learning and memory
Bilateral ovariectomy + MPTP (30 mg/kg/day, 6 times, i.p.)	Rodent	63	5	• Shuffling gait as PD • Loss balance and coordination in rotarod test • Loss muscle strength	• MWM • Y-maze	Deficits in working memory and spatial memory
Maternal separation (3 h/day, 13 or 14 days) + 6-OHDA (5 μg, MFB)	Rodent	2	• 14 • 73	Forelimb use asymmetry	MWM	Deficits in spatial learning and memory
MPTP (25 mg/kg/3.5 days, 10 times, i.p.) + narrowing of the bilateral common carotid arteries	Rodent	56–70	63	Loss motor coordination in the climbing pole	MWM	Spatial learning and memory deficits
Rotenone (12 μg/side, SNpc) + sleep deprivation (24 h or 6 h/day, 21 days)	Rodent	• 49 • 90	• 2 • 47	Hypolocomotion	NOR	Deficit in recognition memory and human episodic-like memory
Rotenone (2 mg/L, 4 weeks) + sleep deprivation (24 h)	Zebrafish	120	28	Hypolocomotion	NOR	Deficit in recognition memory

*6-OHDA, 6-hydroxy dopamine; MPTP, 1-methyl-4-phenyl-1,2,3,6-tetrahydropyridine; NOR, novel object recognition test; MWM, Morris water maze task.*

There are currently six kinds of dual/multiple toxin-induced models of PD with cognitive deficits (Table 6). 6-OHDA- and LPS-combined lesions may provide a new model for verifying associations between memory process vulnerabilities and the stress reactivity of the temporal brain in PD (Hritcu et al., 2011). The hypocretin/orexin (HO) system plays a central role in memory processes. The model induced by 6-OHDA and saporin anti-orexin-B showed deficits in long-term and spatial memory, but not short-term and working memory, which suggests that the HO system is functionally involved in memory processes and consolidation in PD (Oliveira et al., 2020). A PD-MCI model induced by paraquat and maneb administered twice a week for a total of 12 times was characterized by deficits in recognition memory, spatial learning, and memory by damaging synapses in the hippocampus and DA neurons in the SN (Ding et al., 2020). It is a relatively mature combined model that has been widely used in pre-clinical studies of potential therapies for PD-MCI (Wang et al., 2021). On this basis, *N*-(2-chloroethyl)-*N*-ethyl-2-bromobenzylamine (DSP-4), which damages locus coeruleus/norepinephrine neurons and depletes norepinephrine, combined with paraquat and maneb, could exacerbate spatial learning and memory *via* ferroptosis and microglia-mediated neuroinflammation (Hou et al., 2019). Moreover, the model induced by DSP-4 and 6-OHDA developed a similar phenotype (Rampersaud et al., 2012). An LPS and recombinant monomeric human α-Syn co-induced model that has the pathologic features of LB-formation and neuroinflammation could potentiate the effects of each vector and may be a high potential model for studying PD-MCI and PDD (La Vitola et al., 2021).

There are currently 12 kinds of models that combine toxins and transgenes (Table 7). Because both genetic makeup and environmental factors have a crucial role in the early onset of PD, these combined rodent models are useful tools for assessing the effects of environmental factors on genetically susceptible individuals. The combination of one kind of toxin, such as 6-OHDA, MPTP, reserpine, α-Syn oligomers, human α-Syn preformed fibrils, and LPS, and one kind of transgenic mouse, including APP_swe_/PS1△E9 mice, CHT^HET^ mice, mice expressing the human E4 isoform, hTau mice, TKO mice, Nestin-GFP mice, hD1 mice, P*rnp*^0/0^ mice, TLR4^–/–^ mice, 5xFAD Tg mice, and A53T mice resulted in memory, attention, and motor defects (Zurkovsky et al., 2013; Melief et al., 2015; Klein et al., 2016; Bruns et al., 2018; La Vitola et al., 2018, 2019, 2021; Gratuze et al., 2019; Bassil et al., 2020; Torres et al., 2020). These models, which are more closely related to the pathogenesis of PD-MCI and PDD, may be more suitable for assessing the efficacy of a therapeutic agent. However, as generating models that combine a toxin and transgene is difficult, cumbersome, and cost-consuming, significant additional research and discussions are required.

Six kinds of models combined a toxin and surgery to understand the impact of the relationship between risk factors and cognitive deficits in PD, yielding a total of 10 articles (Table 8). For instance, the model combining a toxin (6-OHDA or MPTP) and a bilateral ovariectomy replicated PD-MCI and PDD phenotypes, indicating that parkinsonism aggravates during the premenstrual period in the setting of the lowest levels of estrogen and progesterone (Arbabi et al., 2016; Yadav et al., 2017). Maternal separation, a method that imitates a state of stress, worsens cognitive function in a PD model (Dallé et al., 2017). A model that combined MPTP and narrowing of the bilateral common carotid arteries explored the relationship between orthostatic hypotension and cognitive impairment in PD, reporting that hypotension aggravates cognitive impairment in PD by exacerbating hippocampal damage and white matter lesions. Finally, a model combining rotenone and sleep deprivation reported that the combination of pesticide exposure and insomnia is a key risk factor for cognitive deficits in PD (Gao et al., 2019; Kmita et al., 2019; Lv et al., 2019).

## Considerations

Various experimental models for PD-MCI or PDD have been generated using multiple methods and diverse animal species. Unfortunately, there is no perfect experimental model for studying PD-MCI or PDD, and five pivotal problems that are summarized in the proceeding paragraphs require further consideration and resolution.

One principal challenge is that the difference between PD-MCI and PDD has not been standardized in an experimental model, although there are analogous phenotypic, behavioral, biochemical, and anatomical impairments that represent both PD-MCI and PDD. A clear distinction between PD-MCI and PDD will help transform the results of experimental studies into clinical applications more easily. Based on the diagnostic criteria of PD-MCI and PDD, the severity of cognitive impairment is a key distinction between these pathologies (Dubois et al., 2007; Litvan et al., 2012). A PD model could be used as a PD-MCI model if any one of the following cognitive impairments appears: attention, executive function, memory, or visuospatial function as assessed using different kinds of behavioral tests. When the model has two or more of the above cognitive deficits, it can be considered a model of PDD. Importantly, researchers need to understand the various testing methods that are used to assess PD symptoms, such as dyskinesia and cognitive impairment, from different perspectives. In assessing dyskinesia, the foot-printing test, the rotarod test, the pole test, and the balance beam are commonly used and authoritative methods in rodents. Among these, the balance beam, a simple and direct balance and motor coordination test, is superior to the rotarod test for testing fine movement defects. For NHP, we can assess dyskinesia based on the Unified Parkinson’s Disease Rating Scale. Moreover, we can evaluate the motor function of the drosophila or zebrafish by analyzing their movements paths. For single-sided injections, such as 6-OHDA, specific stereotyped movements after injection of apomorphine is an important test for measuring dyskinesias (Smith et al., 2017; Real et al., 2019). As the DA content in the striatum of the injured side decreases, the number and sensitivity of DA D2 receptors increase significantly. Apomorphine, a DA receptor agonist, can make the excitatory effect of the injured side dominate, thereby inducing the spontaneous rotational movement of models. In addition, VDR, CPT, MDR, SDR, ASST, MWM, NOR, PAT, Barnes maze test, T-maze test, and Y-maze test are the authoritative and representative methods for measuring cognitive impairment. Among these, MWM, a common test to investigate spatial learning and memory in rodents, can be used to assess lesions in distinct brain regions, such as the hippocampus, striatum, cerebral cortex, basal forebrain, and cerebellum (D’Hooge and De Deyn, 2001). In addition to detecting spatial learning and memory functions, the Y maze can detect the ability of animals to recognize spatial position and orientation, the ability to avoid conditioned reflexes, spatial working memory, and fragmented memory. The Barnes maze test is used to evaluate spatial memory and non-spatial memory of experimental animals and can distinguish reference memory from working memory. NOR, a fine and sensitive behavioral method, uses rodents’ instincts to approach and explore novel objects to detect recognition and memory. Thus, we should select the corresponding method based on the different experimental requirements, conditions, and objects. This also requires the evaluation of motor and cognitive function in novel models using different types of behavioral experiments. Forming a set of plans that integrate symptoms, biochemistry, neuroimaging, and other objective indicators to judge and identify the success of a proposed novel model is therefore needed.

An additional challenge is that most novel models simply develop a cognitive deficit that is measurable by a single or analogous behavioral test in the setting of changes in pathology. No model has been integrated to evaluate phenotypes, such as motor deficits, cognitive impairment, and biomarkers in biofluids, and neuroimaging. It is therefore difficult to determine whether a model represents PD-MCI or PDD. The lack of a comprehensive evaluation tool is a principal obstacle to the mutual transformation of research results from basic experiments to clinical trials, especially with respect to clinical examination and radiology. For example, the CSF of rodents, drosophila, and zebrafish is difficult to obtain. MRI and PET instruments for animals are rare and expensive. However, these problems will be solved in the future with the development of technology. Thirdly, molding rate and mortality, which are significant indicators of the repeatability and stability of a novel model, are rarely mentioned. One example is a work that identified a 60%–80% molding rate in 6-OHDA-induced models (Marshall et al., 2019). Some MPTP-induced models also appeared to have normal cognitive function (Gao et al., 2019). However, the combination of MPTP and pesticides resulted in a high mortality rate in rodents. Mortality would be expected to increase in combined experimental models, and the molding rate and mortality of such models should be calculated in future studies.

Fourth, NHP, the closest species to humans, developed cognitive deficits in MPTP-treated and α-Syn transgenic models. However, the mechanism behind the cognitive impairment that results from these models is unclear (Schneider and Kovelowski, 1990; Vezoli et al., 2011; Niu et al., 2015). Moreover, handling and breeding NHP processes are difficult, time-consuming, and expensive. Thus, how to effectively use NHP for PD research remains a significant issue. We could observe the dynamic transition process from PD-MCI to PDD in NHPs, such as symptoms, biomarkers in biofluids, and neuroimaging, and provide new objective evidence for distinguishing PD-MCI from PDD. Furthermore, in addition to the striatum, SN, and hippocampus, researchers need to pay attention to lesions of the PFC, amygdala, anterior commissure, locus coeruleus, and putamen in the NHP model. Importantly, we need to use a neural network perspective to study the pathological mechanisms of PD-MCI and PDD in the NHP model, given their complex pathogenesis (Gratwicke et al., 2015). Finally, cellular models are necessary for mechanism research but have not been used in studies on PD-MCI or PDD. Abnormal aggregation of α-Syn and human α-Syn oligomers were positively correlated with the severity of cognitive deficits in PD (Finkelstein et al., 2016; Liu L. et al., 2020). Inducible α-Syn overexpression and SH-SY5Y-A53T cellular models are good candidate models of PD-MCI or PDD (Zhang et al., 2020; Zhu et al., 2021). However, it is currently difficult to distinguish PD-MCI from PDD at the cellular level.

## Conclusion

While each of the models discussed in the present work has their own disadvantages, there have been a tremendous number of advances in PD cognitive impairment models. Almost every model can verify a hypothesis about PD-MCI or PDD. Based on previous research, we identified a set of plans that integrate symptom, biochemistry, neuroimaging, and other objective indicators to judge and identify that the novel model plays a key role in new strategies for developing representative models. Moreover, we may develop representative models based on the different clinical features of PD-MCI and PDD. Simultaneously, inducible α-Syn overexpression and SH-SY5Y-A53T cellular models and good candidate models of PD-MCI or PDD are important directions for the development of novel models. With the development of improved technology and in-depth research methods, novel models will become increasingly in line with the complex neurodegenerative symptoms and mechanisms of PD-MCI and PDD.

## Author Contributions

YF and MZ designed the research. YF and JH analyzed the data. YF and LZ wrote and reviewed the manuscript. ZH, XH, and YJ reviewed the manuscript. XH, CW, and PW edited figures and tables. DC and MZ supervised the research and reviewed the manuscript. All authors contributed to the article and approved the submitted version.

## Conflict of Interest

The authors declare that the research was conducted in the absence of any commercial or financial relationships that could be construed as a potential conflict of interest.

## Publisher’s Note

All claims expressed in this article are solely those of the authors and do not necessarily represent those of their affiliated organizations, or those of the publisher, the editors and the reviewers. Any product that may be evaluated in this article, or claim that may be made by its manufacturer, is not guaranteed or endorsed by the publisher.

## Abbreviations

PD: Parkinson’s disease
PD-MCI: Parkinson’s disease mild cognitive impairment
PDD: Parkinson’s disease’s disease dementia
α-Syn: α-synuclein
LBs: Lewy bodies
AD: Alzheimer’s disease
SN: substantia nigra
LRRK2: leucine-rich repeat serine/threonine-protein kinase 2
MRI: magnetic resonance imaging
PET: positron emission tomography
DAergic: dopaminergic
MPTP: 1-methyl-4-phenyl-1,2,3,6-tetrahydropyridine
6-OHDA: 6-hydroxy dopamine
MFB: medial forebrain bundle
PFC: prefrontal cortex
VTA: ventral tegmental area
NHP: non-human primates
VDR: variable delayed response task
CPT: continuous performance task
NOR: novel object recognition test
MWM: Morris water maze task.
