# Corrigendum: Experimental Models of Cognitive Impairment for Use in Parkinson's Disease Research: The Distance Between Reality and Ideal

**DOI:** 10.3389/fnagi.2021.827753

**Published:** 2021-12-21

**Authors:** Yaohua Fan, Jiajun Han, Lijun Zhao, Chunxiao Wu, Peipei Wu, Zifeng Huang, Xiaoqian Hao, YiChun Ji, Dongfeng Chen, Meiling Zhu

**Affiliations:** ^1^Traditional Chinese Medicine Innovation Research Center, Shenzhen Hospital of Integrated Traditional Chinese and Western Medicine, Guangzhou University of Chinese Medicine, Shenzhen, China; ^2^Guangzhou University of Chinese Medicine, Guangzhou, China; ^3^Shenzhen Bao'an Traditional Chinese Medicine Hospital, Guangzhou University of Chinese Medicine, Shenzhen, China

**Keywords:** Parkinson's disease, cognitive impairment, experimental model, toxins, transgenic animal, limitations

In the published article, there was an error regarding the affiliation**s** for **Dongfeng Chen and Meiling Zhu**. As well as having affiliation **1, Dongfeng Chen should also have 2. As well as having affiliation 2, Meiling Zhu should also have 1**.

The authors apologize for this error and state that this does not change the scientific conclusions of the article in any way. The original article has been updated.

## Publisher's Note

All claims expressed in this article are solely those of the authors and do not necessarily represent those of their affiliated organizations, or those of the publisher, the editors and the reviewers. Any product that may be evaluated in this article, or claim that may be made by its manufacturer, is not guaranteed or endorsed by the publisher.

